# Patients with low back pain differ from those who also have leg pain or signs of nerve root involvement – a cross-sectional study

**DOI:** 10.1186/1471-2474-13-236

**Published:** 2012-11-28

**Authors:** Alice Kongsted, Peter Kent, Hanne Albert, Tue Secher Jensen, Claus Manniche

**Affiliations:** 1 Research Department, The Spine Centre of Southern Denmark, Hospital Lillebaelt, Institute of Regional Health Services Research, University of Southern Denmark, Clinical Locomotion Science Network, Middelfart, Denmark

**Keywords:** Classification, Cohort studies, Low back pain, Radiculopathy, Sciatica

## Abstract

**Background:**

Leg pain associated with low back pain (LBP) is recognized as a risk factor for a poor prognosis, and is included as a component in most LBP classification systems. The location of leg pain relative to the knee and the presence of a positive straight leg raise test have been suggested to have clinical implications. To understand differences between such leg pain subgroups, and whether differences include potentially modifiable characteristics, the purpose of this paper was to describe characteristics of patients classified into the Quebec Task Force (QTF) subgroups of: 1) LBP only, 2) LBP and pain above the knee, 3) LBP and pain below the knee, and 4) LBP and signs of nerve root involvement.

**Methods:**

Analysis of routine clinical data from an outpatient department. Based on patient reported data and clinical findings, patients were allocated to the QTF subgroups and described according to the domains of pain, activity limitation, work participation, psychology, general health and clinical examination findings.

**Results:**

A total of 2,673 patients aged 18–95 years (median 47) who were referred for assessment of LBP were included. Increasing severity was consistently observed across the subgroups from LBP only to LBP with signs of nerve root involvement although subgroup differences were small. LBP patients with leg pain differed from those with LBP only on a wide variety of parameters, and patients with signs of nerve root involvement had a more severe profile on almost all measures compared with other patients with back-related leg pain.

**Conclusion:**

LBP patients with pain referral to the legs were more severely affected than those with local LBP, and patients with signs of nerve root involvement were the ones most severily affected. These findings underpin the concurrent validity of the Quebec Task Force Classification. However, the small size of many between-subgroup differences amid the large variability in this sample of cross-sectional data also underlines that the heterogeneity of patients with LBP is more complex than that which can be explained by leg pain patterns alone. The implications of the observed differences also require investigation in longitudinal studies.

## Background

Low back pain (LBP) is commonly triaged or classified into LBP due to serious pathology, LBP with nerve root compression, and non-specific LBP (NSLBP) [1]. Most patients with NSLBP and many with nerve root compression are treated with conservative care, but the demonstrated effect sizes are often small, sometimes moderate and important differences in effects between interventions have not been convincingly demonstrated [2-5].

The belief that NSLBP consists of a number of subgroups, with different prognoses and different treatment responses, has increased over the last decade, and this has been mirrored in a proliferation of studies reporting research into NSLBP subgroups aimed at identifying homogeneous groups of patients with similar trajectories or who would benefit from a certain intervention [6-10]. It is well-established that patients with leg pain in addition to LBP have a poorer prognosis than patients with local LBP only [11] and leg pain is a component of most LBP classification tools [12-15]. However, the LBP patient group with leg pain has been defined in diverse ways, from those with any leg pain to those with radiculopathy and an MRI-confirmed clinical diagnosis of nerve root compression [16].

The report of the 1987 Quebec Task Force suggested distinguishing between pain referral above and below the knee [17]. This has since been shown to be associated with prognosis [18,19], and a recent study in primary care showed that patients with leg pain referred above or below the knee differed on symptom severity as well as psychological characteristics compared with each other and compared with patients who had no pain referral. Importantly, this primary care study also showed that these baseline differences explained most of the variability in outcomes [20]. Other studies have shown that patients with distal leg pain and a positive straight leg test had more severe symptoms, heavier psychological distress and more health care utilization than those without a positive straight leg raise [21,22]. One application of knowledge of such potentially modifiable characteristics associated with different leg pain patterns is that they may provide direction for the development of treatments targeted to different types of radiating leg pain.

Put simply, knowing the distinctions between patient groups with LBP only, those also with leg pain above the knee, those with leg pain below the knee and those with nerve root involvement, would clarify which subgroup classifications are useful. This information could assist in clinical decision making for individual patients, in quality assurance and in the design of research projects.

The objective of this study was to describe physical, psychological and social factors in LBP patients when they first had contact with a secondary care outpatient clinic. The cohort was described using the Quebec classification subgroups of (a) LBP only, (b) LBP and leg pain above the knee, (c) LBP and leg pain below knee, and (d) LBP and signs of nerve root involvement.

## Methods

### Setting

This study was based on data collected as part of the daily clinical routine in the conservative care department of The Spine Centre of Southern Denmark. This outpatient, secondary care department is a non-surgical unit in a public hospital with a geographic catchment area with approximately 1.2 million inhabitants [23]. The department principally performs multidisciplinary assessment of patients with spinal pain after referral from general practitioners, chiropractors, and medical specialists in primary care. A structured clinical examination and use of MRI are core elements, and short courses of conservative treatment can be offered to test a patient’s response to treatment.

### Data collection

Data were collected in the Spine Centre’s electronic clinical registry named the SpineData database (Regional Ethics Committee Project ID S-200112000-29). Patients answered a comprehensive self-reported baseline questionnaire on a touch screen in the waiting area prior to their first consultation. The clinicians entered results of a core set of clinical tests either when examining the patient or after the first consultation. Clinicians in the department were physical therapists, chiropractors, rheumatologists, orthopaedic surgeons and general medical practitioners.

### Study sample

Patients aged 18 years or older who were seen in The Spine Centre between December 15^th^ 2008 and November 4^th^ 2011 with LBP as their main complaint were selected for the analyses. The main complaint was defined by the patient’s response when asked to indicate on the touch screen body chart the area for which the patient was seeking care. To be included in the study, patients had to have completed the Centre’s electronic patient questionnaire with no missing data on pain intensity or in the pain drawing. An additional inclusion criterion was that data from a clinician’s neurological examination needed be present. For patients who were referred to the centre for more than one episode of LBP during the study period (< 2% of the cohort) only data for the latest episode was included.

### Health domains

The cohort was described on six health domains (pain, activity limitation, work participation, psychological factors, quality of life, and clinical examination). Except for the clinical examination findings, all items were self-reported by patients.

Pain items were: duration of the present episode (months), previous LBP episodes (yes/no), intensity of low back pain (score on 0-10 Numerical Rating Scale (NRS) for each of ‘LBP now’ , ‘worst in the last 14 days’ , ‘typical in the last 14 days’ , which were collectively averaged to form a single 0-10 scale) [24], intensity of leg pain (measured in the same way as for LBP), severe leg pain (intensity of leg pain > 3) [25], dominating leg pain (proportion reporting higher intensity of leg pain than intensity of LBP), and pain irritability (requiring a yes-answer to both’ pain is easily aggravated by physical activity’ and’ it takes a long time before it settles again’) [26,27].

Activity limitation was measured with the 23-item Roland Morris Disability Questionnaire (RMDQ) [28] and calculated as a proportional score (0% = no activity limitation; 100% = maximum activity limitation) [29].

Work participation was assessed by questions on: Regular employment (proportion of the working population, i.e. not studying or retired, being employed without public benefits), sick listing (proportion of persons in regular employment who reported any sick listing within the previous three months), and sick days (days off work during the preceding three months among the regular employed).

Psychological characteristics were depressive symptoms and fear of movement. Depressive symptoms were measured by the two PRIME-MD 1000 screening questions [30] using a 0–10 NRS (proportion of patients with a score above 6 on both questions). These cut-points were derived in an unpublished study in our patient setting based on a comparison with population-based thresholds for the Beck Depression Index [31] and the Major Depression Inventory [32]. Pain-related fear of movement was measured using NRS 0-10 scales (proportion with a total score on two screening questions from the Fear Avoidance Belief Questionnaire equal to or above 14) [33]. This threshold was also derived in an unpublished study in our patient setting based on a comparison with a primary care score threshold (mean plus 1 standard deviation) on the physical activity subscale of the Fear Avoidance Belief Questionnaire.

General health was assessed using the Euroqol health thermometer (Euroqol VAS) that measures self-reported health state today (0 = worst imaginable; 100 = best imaginable) [34].

Directional preference was recorded from the clinical examination and defined as being present if either centralisation or peripheralisation of pain occurred with tests of end-range movements or postures as described in the Mechanical Diagnosis and Therapy system by McKenzie [15]. Centralization has been defined as “the abolition of distal pain in response to the deliberate application of movements or postures. If pain is only in the back this is centralized and then abolished” [35]. Peripheralisation is the opposite pain response to movements or postures.

### Definitions of subgroups

Patients were classified as having *local LBP only* if their pain drawing only included local LBP and their worst leg pain intensity in the preceding 14 days was zero (0-10 scale). LBP with leg pain above the knee *(LBP + pain above knee)* was defined as persons who indicated on the pain drawing that they had pain in the anterior or posterior thigh but no pain in the calf or feet, and rated their worst leg pain intensity as being one or more (0-10 scale). LBP with leg pain below the knee *(LBP + pain below knee)* was defined as a person with a pain drawing indicating pain in the calf and/or foot and their worst leg pain intensity being at least one. LBP with signs of nerve root involvement *(LBP + NRI*) was arbitrarily defined as any person with any leg pain present on their pain drawing, their worst leg pain intensity was one or more, and at least one of the following findings was present on the painful side during the clinical examination: muscle weakness, impaired tendon reflexes, altered sensation to touch or pinprick, a straight leg raise test that provoked their familiar leg pain (at 60 degrees or less as judged visually), or a positive prone knee bend test combined with pain to the anterior thigh (Reverse Laségue Test). The term ‘signs of nerve root involvement’ should be considered a label given to patients fulfilling these criteria rather than a definition of a diagnostic entity. There is always imprecision for the classification of radiculopathy when only clinical signs are available [36] and our necessarily arbitrary definition was a pragmatic choice using the clinical findings available within a routine care setting. In this *LBP + NRI* subgroup single signs of NRI occurred as a positive SLR in 117 patients, altered sensation in 149, muscle weakness in 3 patients, and impaired tendon reflexes in 4, while the rest of the group had two or more signs.

Patients, whose pain drawing and pain scales were ambiguous, for example where their leg pain intensity was reported as zero but they indicated leg pain on the pain drawing, were excluded from the analysis. People excluded for this reason did not necessarily give contradictory or inaccurate answers. For example, a person could have reported having leg pain within the previous two weeks but did not have leg pain on the exact day when the pain drawing was made. However, allocation of such a person into a classification group would have been ambiguous and we opted to exclude such patients in order to obtain the most definitive data.

### Data analyses

Results are reported either as proportions with 95% confidence intervals (95% CI) or medians with inter-quartile ranges (IQR) (since most continuous variables were not normally distributed). Differences between the subgroups were tested using Kruskal-Wallis one-way Analysis of Variance. When group differences were significant at the 5% level, pairwise comparisons were performed using Chi Square Tests for proportions and, *t*-test or Wilcoxon Ranksum Test for ordinal and continuous variables. Group differences were considered statistically significant at p<.05. As the high number of variables tested increases the risk of mass significance, between-subgroup comparisons should be interpreted as only hypothesis-generating. Analyses were performed using STATA IC 11.2 (StataCorp, Texas, USA).

## Results

### Study cohort

**Figure 1:**
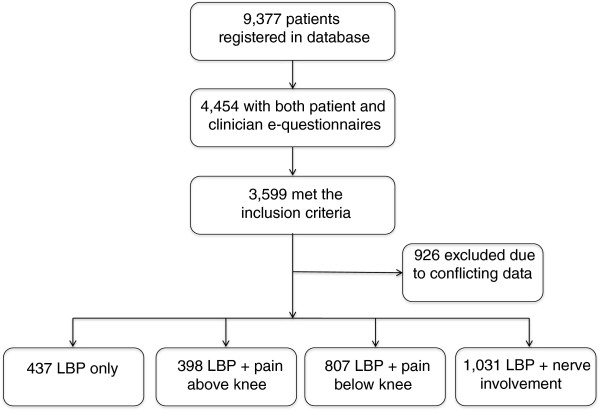
Flow of patients from entering the department to allocation to subgroups in the study.

A total of 9,377 people above 18 years were registered in the database as new LBP patients during the study period. Due to a staged implementation of the electronic questionnaires across the four geographic sites of The Spine Centre during this period, there were both patient and clinician questionnaires available in electronic format for only 4,294 of them. People completing the electronic questionnaire had the same gender distribution as the entire patient population and were on average one year younger. We excluded 964 people from the analyses because of ambiguous data. After excluding such patients and those with missing data, 2,673 people fulfilled the criteria for allocation to subgroups. The study flowchart is shown in Figure 1. Those allocated to the subgroups had worse leg symptoms and slightly more activity limitation as compared with patients who could not be categorised into the subgroups (Table 1). A detailed description of the whole cohort and the subgroups is shown in Table 2.

**Table 1:** A comparison between those patients allocated to study subgroups and patients who could not be allocated to subgroups because of ambiguous data

	**Allocated to subgroups n = 2,673**	**Registered patients not allocated to subgroups n=926**
Females, % (95% CI)	56 (54-58)	54 (51-57)
Age in years, mean (SD)	48 (15)	48 (15)
Duration > 12 months, % (95%CI)*	47 (45-49)	52 (49-55)
LBP intensity (0-10), median (IQR)	6 (4-8)	6 (5-7)
Leg pain intensity (0-10), median (IQR)*	5 (3-7)	3 (1-6)
Leg pain > 0 (0-10), % (95% CI)*	84 (83-86)	88 (86-90)
Signs of nerve root involvement, % (95%CI)*	40 (38-42)	30 (27-33)
Activity limitation (0-100), median (IQR)*	65 (43-82)	61 (39-78)

CI: Confidence interval.

* p<.05.

**Table 2:** Characteristics of patients with low back pain and of patients who also have leg pain or signs of nerve root involvement

	**All subgroups**	**Local LBP only**	**LBP + pain above knee**	**LBP + pain below knee**	**LBP + signs of nerve root involvement**	**p-values**
	**n = 2,673**	**n = 437**	**n = 398**	**n = 807**	**n = 1,031**	**Significant pair-wise comparisons**
**Females,** (95% CI)	56% (54%-58%)	49% (44%-54%)	59% (54%-64%)	60% (57%-64%)	54% (51%-57%)	p < .01 local vs. above local vs. below below vs. NRI
**Age in years,** median (IQR)	47 (36-58)	43 (32-55)	46 (35-58)	50 (39-63)	46 (37-58)	p < .001 local vs. all below vs. all
Duration*, (95% CI) **0 – 3 months**	18% (16%-19%)	8% (6%-11%)	13% (10%-16%)	15% (12%-17%)	27% (24%-29%)	p < .001 local vs. below NRI vs. all
**3 – 12 months**	34% (32%-36%)	34% (30%-39%)	36% (31%-41%)	35% (31%-38%)	35% (32%-38%)	
**> 12 months**	45% (44%-47%)	57% (53%-62%)	51% (46%-56%)	51% (47%-54%)	38% (35%-41%)	
**Previous episodes,** (95% CI)	74% (73%-76%)	67% (62%-71%)	77% (72%-80%)	76% (72%-79%)	79% (76%-81%)	p < .01 local vs. all
**Pain irritability,** (95% CI)	74% (72%-76%)	60% (53%-66%)	73% (67%-78%)	76% (72%-80%)	79% (75%-82%)	p < .001 local vs. all
**LBP intensity,** median (IQR)	6 (5-8)	5 (4-7)	6 (4-7)	6 (4-8)	6 (5-8)	p < .001 all comparisons except above vs. below
**Leg pain intensity,** median (IQR)	3 (5-7)	0 (0-0)	4 (3-6)	6 (4-7)	6 (5-8)	p < .001 all comparisons
**Severe leg pain,** (95% CI)	70% (68%-72%)	0 (0-0)	68% (64%-73%)	85% (82%-87%)	88% (86%-90%)	p < .001 all comparisons
**Dominating leg pain,** (95% CI)	29% (27%-31%)	0 (0-0)	23% (18%-27%)	36% (32%-39%)	38% (35%-41%)	p < .001 all comparisons except NRI vs. below
**Activity limitation,** median (IQR)	65 (43-83)	48 (26-67)	57 (39-74)	65 (43-78)	74 (52-87)	p < .001 all comparisons
**Regular employment**^**§**^ (95% CI)	66% (64%-68%)	77% (73%-82%)	65% (60%-71%)	62% (58%-66%)	66% (63%-70%)	p = .01 local vs. all
**Any sick leave in last 3 months**^**#**^**,** (95% CI)	49% (46%-51%)	41% (35%-47%)	44% (36%-52%)	47% (41%-52%)	56% (52%-61%)	P <.001 NRI vs. all
**Sick leave days in last three months**^**#**^**,** median (IQR)	14 (5 – 30)	10 (4 – 25)	10 (5 – 21)	14 (5 – 34)	18 (7-34)	< .01 local vs. below local vs. NRI
**Depressive symptoms,** (95% CI)	17% (16%-19%)	12% (9%-15%)	16% (12%-19%)	16% (14%-19%)	21% (18%-23%)	p < .001 local vs. below NRI vs. all
**Fear of movement,** (95% CI)	19% (18%-21%)	15% (12%-19%)	16% (12%-19%)	18% (16%-22%)	23% (20%-25%)	P=.06 NRI vs. all
**General health,** median (IQR)	50 (30-70)	53 (40-75)	50 (33-70)	50 (30-67)	48 (29-64)	p < .001 local vs. all above vs. NRI
**Directional preference,**^**¤**^ (95% CI)	36% (33%-39%)	19% (13%-25%)	32% (24%-39%)	31% (26%-37%)	49% (44%-54%)	p < .001 all comparisons except above vs. below

95% CI = 95% confidence interval; IQR = inter quartile range; NRI = Nerve root involvement signs.

^*^Does not sum to 100% because of missing values.

^#^Proportion of patients in regular employment.

^¤^Proportion of patients with a test conclusion n=1,012(38%)/159(36%)/168(42%)/283(35%)/402(39%.)

The included cohort consisted of 56% females and was aged 18-95 years (median 47 years). Patients with *local LBP only* and those with *LBP + NRI* were less often females than patients with *LPB + pain above knee* and *LBP + pain below knee*, whereas the median age of patients in the *LBP + pain below knee* subgroup was higher than in the other groups (Table 2). Of the patients allocated to the *LBP + pain below knee* subgroup, 52% had pain also in the foot according to their pain drawing, and for those with *LBP + NRI* this was the case in 55% of the participants*.*

### Pain

**Figure 2:**
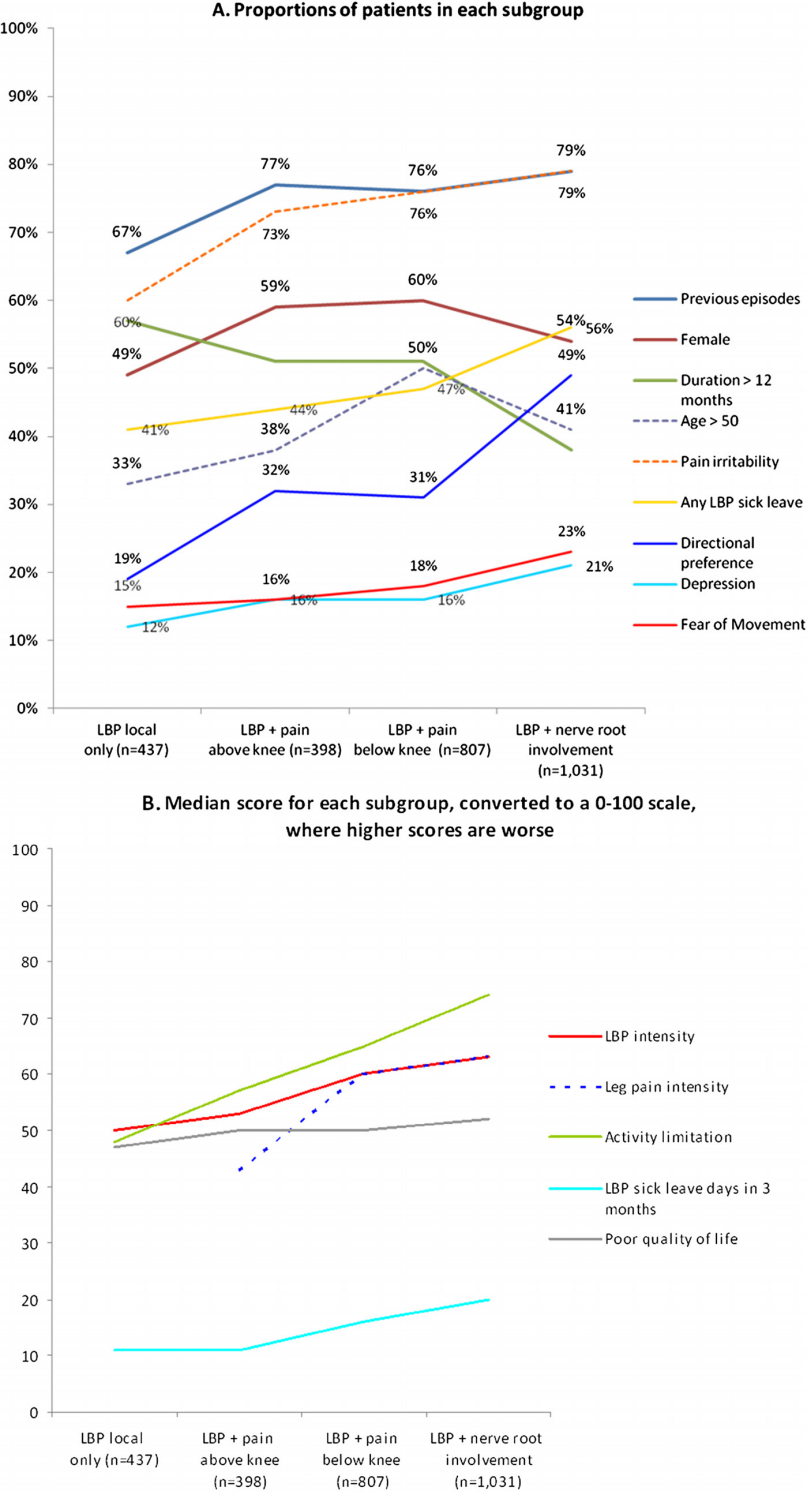
Trends across subgroups on all measured health parameters converted to a 0-100 scale.

Patients with *local LBP only* had the longest duration of pain at the time of first consultation, and those with *LBP + NRI* the shortest duration. About three-quarters of the cohort reported previous LBP episodes, which were less frequent in the *local LBP only* group as compared with patients with leg pain (Table 2). Pain irritability was also common in all groups but more in the subgroups with leg pain. LBP intensity was similar across subgroups whereas patients in *LBP + pain above knee* reported less intensive leg pain than those with *LBP + pain below knee* and *LBP + NRI*. Within the pain domain there was a general trend of worsening from the *local LBP only* to the *LBP + NRI* group on all parameters (Figure 2A and B).

### Activity limitation

The median RMDQ proportional score was 65 (Table 2). There was a trend for more activity limitation across the groups from *local LBP only* having the least, to the *LBP + NRI* group, which had the most activity limitation (Figure 2B).

### Work participation

Sixty-six percent of patients belonging to the working population were in regular employment with higher work participation in the group with *local LBP only* than in the leg pain subgroups. Around half of those working had been sick-listed within the previous three months with more frequent sick listing and the highest number of sick leave days reported in the *LBP + NRI* group (Table 2).

### Psychological factors

Symptoms of depression and fear of movement were most frequently reported among patients with *LBP + NRI* and were least frequently reported in the *local LBP only* patients (Table 2 and Figure 2A).

### General health

Self-reported general health was of almost the same magnitude in all groups, although the highest score was observed in the *LBP only* group and the lowest in the group with *LBP + NRI* (Table 2).

### Clinical examination

Less than half of the population was tested for directional preference, from 36% of *LBP only* patients to 42% of patients with *LBP + pain above knee*. A directional preference was present in about 50% of tested patients with *LBP + NRI* and in less than 20% with *local LBP only*. The proportion of patients with a directional preference did not differ between the groups with *LPB + pain above knee* and *LBP + pain below knee* (Table 2). One reason that all patients were not tested for directional preference was that not all clinicians were trained in the Mechanical Diagnosis and Therapy system.

An additional finding within the clinical examination domain was that 51% of all patients with pain below the knee and 32% of patients with leg pain only above the knee had sign of nerve root involvement.

## Discussion

This study described patients with LBP seen in a secondary care outpatient department using four predefined subgroups based on the Quebec Task Force classification [17]. Overall, the differences observed between these groups were small. However, people who had local LBP only differed from those in the leg pain subgroups on almost all the described characteristics. Furthermore, there was a consistent trend in all health domains of increasing severity from the subgroup with local pain, across the subgroups with pain referral above and below the knee, to the subgroup with signs of nerve root involvement.

Different patient profiles were observed among patients with leg pain depending on whether they had signs of nerve root involvement or not. Patients with signs of nerve root involvement had a more severe profile on measures from all health domains. Similar findings were observed in a previous study using the Quebec Task force classification [19], and in studies that demonstrated that the presence of a positive straight leg raise identified patients with a characteristic profile [21,22].

Comparing people with pain above the knee and those with pain below the knee revealed higher leg pain intensity and more activity limitation in the group with more distal pain. There were little differences in other parameters according to location above and below the knee, although generally patients with *LBP + pain below knee* in this secondary care setting tended to be more severely affected than those with *LBP + pain above knee* as previously observed in other settings [20,21].

LBP with any leg pain tended to be more severe than local LBP only, regardless of how severity was measured, and was also associated with higher frequencies of psychological risk factors. This does not clarify any causal direction and it may simply be that more distal pain actually is experienced as more severe and results in heavier psychological distress, and therefore these are mutually interdependent covariates. The relationship between pain and depression was demonstrated to be reciprocal in a study by Kroenke [37], but the potential causative influence of leg pain on psychological risk factors or the reverse needs to be investigated, including potential differences in this relationship between leg pain subgroups.

The main strength of this study was the large sample size which is likely to produce more trustworthy estimates. Moreover, we expect the results to be generalisable to other secondary care settings since data were collected as part of the everyday routine of the department and not constrained by the strict inclusion criteria that are often necessary in clinical trials. The downside of using registry data is less control of data quality and so, in order to describe subgroups with very clearly defined profiles, we took the approach of excluding patients who reported any ambiguous data. By so doing, we chose to maximise the ‘signal’ in data (clear subgroups) by reducing the ‘noise’ (ambiguous data).

A limitation of this study was the definition of nerve root involvement. Classification into the group with signs of nerve root involvement required the presence of just one positive finding in the neurological examination, and the reliability of these findings in this setting is unknown. The unquantified reliability of our examination procedures is an inherent limitation of data from large clinical databases that were not collected for a specific research project. However, the data were also inherently more representative of routine care and our results demonstrate that even this ‘loose’ definition of signs of nerve root involvement defines a distinctive subgroup. It is possible that our definition of ‘nerve root involvement’ led to underestimation of subgroup differences, but the findings indicated that even very simple clinical examination data can add valuable information.

Another potential weakness of this study is that the inclusion criteria and the gradual implementation of the electronic database over the study period meant that only about 25% of the patients seen during this time period were described in this study. All known selection biases have been reported. There were no clinically important differences on gender or age between those who filled in the electronic questionnaires and those who did not. People who could be unambiguously allocated to the defined subgroups had slightly more severe symptoms and more disability than those who could not be allocated. This bias towards those with a more severe profile means that the proportions allocated to each group may not truly reflect the whole patient population.

A further weakness is that some constructs, notably the psychosocial constructs, were measured using screening questions only, though such an approach has been used in other contexts including in the Bournemouth Questionnaire [38]. The screening questions used had high overall accuracy (86.8% to 88.0%) for predicting the scores on the reference standard questionnaires for depression and fear avoidance [unpublished data]. The main reason for using screening questions is that it is simply not possible in our setting to collect comprehensive psychological data on all patients as part of a multi-domain assessment procedure. Our experience suggests that we are at the limit of responder burden that is acceptable to our patients.

Lastly, it should be recognised that some differences between subgroups may have been a result of referral patterns. For example, patients in the cohort with local LBP only had longer pain duration when seen at the department which was probably because leg pain prompts referral at an earlier stage than local LBP.

## Conclusions

In summary, the findings in this study confirm that leg pain in addition to LBP is an important indicator of a more severe condition than LBP only, almost regardless of health domain. Moreover, we found that clinical signs of nerve root involvement defined a subgroup of people more severely affected than other leg pain patients on activity limitation, work participation and psychological factors. Although between-subgroup differences were small, collectively these findings underpin the concurrent validity of the Quebec Task Force Classification. However, the results also show that variability in these cross-sectional data between individual patients with LBP is more complex than can be simply explained by the presence of leg pain patterns. There is also a need to investigate the clinical importance of the observed subgroups differences in longitudinal outcome studies.

## Competing interests

The authors have no financial or non-financial competing interests to declare.

## Authors’ contributions

AK and PK formed the study idea. All authors were involved in the design of the study, interpretation of data, revision of the manuscript, and gave final approval of the manuscript. AK performed the data analysis and wrote the initial draft of the manuscript.

## Pre-publication history

The pre-publication history for this paper can be accessed here:

http://www.biomedcentral.com/1471-2474/13/236/prepub
